# Flexible hidden Markov models for behaviour-dependent habitat selection

**DOI:** 10.1186/s40462-023-00392-3

**Published:** 2023-06-03

**Authors:** N. J. Klappstein, L. Thomas, T. Michelot

**Affiliations:** 1 School of Mathematics and Statistics, University of St Andrews, St Andrews, UK; 2 Department of Mathematics and Statistics, Dalhousie University, Halifax, Canada

**Keywords:** Habitat selection, Animal behaviour, Step selection functions, Hidden Markov models, Animal movement

## Abstract

**Background:**

There is strong incentive to model behaviour-dependent habitat selection, as this can help delineate critical habitats for important life processes and reduce bias in model parameters. For this purpose, a two-stage modelling approach is often taken: (i) classify behaviours with a hidden Markov model (HMM), and (ii) fit a step selection function (SSF) to each subset of data. However, this approach does not properly account for the uncertainty in behavioural classification, nor does it allow states to depend on habitat selection. An alternative approach is to estimate both state switching and habitat selection in a single, integrated model called an HMM-SSF.

**Methods:**

We build on this recent methodological work to make the HMM-SSF approach more efficient and general. We focus on writing the model as an HMM where the observation process is defined by an SSF, such that well-known inferential methods for HMMs can be used directly for parameter estimation and state classification. We extend the model to include covariates on the HMM transition probabilities, allowing for inferences into the temporal and individual-specific drivers of state switching. We demonstrate the method through an illustrative example of plains zebra (*Equus quagga*), including state estimation, and simulations to estimate a utilisation distribution.

**Results:**

In the zebra analysis, we identified two behavioural states, with clearly distinct patterns of movement and habitat selection (“encamped” and “exploratory”). In particular, although the zebra tended to prefer areas higher in grassland across both behavioural states, this selection was much stronger in the fast, directed exploratory state. We also found a clear diel cycle in behaviour, which indicated that zebras were more likely to be exploring in the morning and encamped in the evening.

**Conclusions:**

This method can be used to analyse behaviour-specific habitat selection in a wide range of species and systems. A large suite of statistical extensions and tools developed for HMMs and SSFs can be applied directly to this integrated model, making it a very versatile framework to jointly learn about animal behaviour, habitat selection, and space use.

**Supplementary Information:**

The online version contains supplementary material available at 10.1186/s40462-023-00392-3.

## Introduction

Wildlife conservation requires an understanding of animal movement and space use [36]. To prioritise areas of conservation interest, it is important to know what habitat features animals use for crucial life processes. These habitat choices (termed ‘habitat selection’) have been studied extensively to understand how animals respond to foraging resources [5], environmental risks (e.g., predators, roads; [15, 38]), and other landscape features or resources (e.g., slope, terrain; [11, 15]). Habitat selection can vary between behaviours, which often require different resources [30]. For example, preferred foraging resources may be different from the habitat features that are best suited for other behaviours, such as travelling or resting. Therefore, models for jointly estimating behaviour switching and habitat selection are needed to delineate critical areas for biologically important behaviours.

Step selection functions (SSFs) are a popular framework to jointly model animal movement and habitat selection [2]. SSFs assess how animals select habitat by comparing the spatial features (e.g., resources and movement metrics) of selected steps to the surrounding environment [2, 14]. Although SSFs are used to analyse time series data covering long time periods, they usually assume that the selection patterns are constant throughout the movement track. It has been shown that pooling habitat selection parameters over periods that span multiple behavioural states can bias estimates [41], but most analyses still ignore this issue. Interactions between movement and habitat covariates can be included to give some insights into how habitat selection varies with movement, but this does not explicitly account for the animal’s behavioural state [2].

Hidden Markov models (HMMs) are commonly used to identify distinct states from animal telemetry data [19, 29]. These states are generally defined based on movement characteristics (e.g., step length or directional persistence), and their dynamics are governed by transition probabilities (e.g., animals may be more likely to stay in their current state than switch). Usually, the estimated states are interpreted as behavioural states, such as resting or travelling (although this will be study-specific; [28]). It is also common to assess how covariates influence the probability of switching between states [19, 31]. This is the most common way to incorporate environmental covariates into an HMM (see examples in [28, 29, 31]), and can further be used to investigate how other temporal (e.g., hour, day of year; [47, 48]) or individual-specific factors (e.g., sex or size; [3]) affect animals’ activity. Although this approach is useful to identify the drivers of behaviours, it falls short of capturing habitat selection directly, as it does not explicitly model individual movement decisions based on habitat features. Therefore, most commonly-used HMMs are inadequate to describe animals’ space use [16].

To assess behaviour-specific habitat selection, some applied studies have employed a two-stage design, in which HMMs are used sequentially with SSFs (e.g., [7, 11, 32, 44]). In this case, the animal path is segmented into discrete behavioural states using an HMM, and segments of each behaviour are jointly analysed with an SSF to produce state-dependent habitat selection parameters. This two-stage approach is convenient as it uses two widely-used methods, both with user-friendly software and literature [23, 28, 43]. However, this approach does not properly account for the uncertainty in behavioural classification, but rather treats the estimated states as data. Further, the state classification is obtained independently of habitat selection. Therefore, the model cannot capture behaviours that are jointly defined by movement and habitat selection, and this may lead to bias or underestimated uncertainty in the habitat selection parameters.

Despite the benefits, it remains complex to analyse habitat selection and behaviour in a unified framework. Nicosia et al. [30] proposed an integrated model that combines an HMM with an SSF (termed the HMM-SSF, also explored further in [37]). The HMM-SSF accounts for multiple sources of uncertainty and has greater flexibility than HMMs or SSFs on their own (and see the preprint by [34], for a full comparison to the two-stage method). The SSF component of the model can be used to estimate both movement and habitat selection parameters, and thereby classifies states based on more information than a typical HMM (with only movement covariates). We write this model as a standard HMM with an SSF governing the observation process, and in this paper, we take advantage of this formulation to implement convenient computational methods and extensions. We propose fitting the model using direct numerical maximisation of the likelihood, based on an efficient iterative algorithm called the forward algorithm. We then extend the model of Nicosia et al. [30] by including covariates on the state transition probabilities, which allows us to estimate effects of external or internal factors on behavioural dynamics. We describe how standard state decoding (i.e., classification into behaviours) can be applied in this context, and show how this spatially-explicit formulation of an HMM can be used to derive space use. Lastly, we present an illustrative analysis of plains zebra (*Equus quagga*) telemetry data as a guide to the application and interpretation of the model. To further make these methods accessible to ecologists, we have provided all necessary R code with the manuscript.

The HMM-SSF model can viewed in two different ways, and we think that each will appeal to many researchers. It can be viewed as an extension of SSFs, with the addition of behavioural switching, which will be useful to biologists who would like to improve their habitat selection inferences and avoid the pitfalls highlighted by Roever et al. [41]. Alternatively, the HMM-SSF can be described as an extension of the HMMs typically used in animal movement ecology, with the inclusion of habitat selection variables (in addition to movement variables such as step lengths and turning angles). This spatial HMM formulation will be more appropriate in cases where practitioners are interested in capturing animals’ space use [16].

## Methods

### Step selection functions

Consider a set of bivariate animal locations with negligible measurement error $\{\varvec{y}_1, \varvec{y}_2, \dots , \varvec{y}_T\}$ at observed times $t = 1, 2, \dots , T$, which can be at any regular interval that is appropriate for the scale of the biological process of interest. An SSF assumes that the probability of an animal taking a given step is determined by both movement constraints and habitat features, such that the likelihood of a step ending at $\varvec{y}_{t+1}$ given that it started as $\varvec{y}_t$ is

1 $$\begin{aligned} p(\varvec{y}_{t+1} \mid \varvec{y}_t) = \frac{w(\varvec{y}_t, \varvec{y}_{t+1}) \phi (\varvec{y}_{t+1} \mid \varvec{y}_t)}{\int _{\varvec{z}\in \Omega } w(\varvec{y}_t, \varvec{z}) \phi (\varvec{z}\mid \varvec{y}_t) d\varvec{z}} \end{aligned}$$

where *w* is a weighting function (evaluated for the step from $\varvec{y}_t$ to $\varvec{y}_{t+1}$) that describes habitat selection, $\phi$ is a movement kernel that models the movement patterns of the animal, and $\Omega$ is the study area [14]. The denominator is a normalising constant, which ensures that the SSF is a probability density function with respect to $\varvec{y}_{t+1}$ [35]. The weighting function *w* is specified as a function of relevant covariates, and the movement kernel $\phi$ is typically used to model distributions of step lengths and turning angles. Both *w* and $\phi$ can take many forms, but as identified by Forester et al. [14] and Avgar et al. [2], it has advantages to specify them both as exponential models (discussed below). Therefore, the entire SSF (which we define as Eq. 1, although the nomenclature is inconsistent in the literature) models the step density, based on both movement and habitat, and represents the relative attractiveness of the selected endpoint $\varvec{y}_{t+1}$ compared to the surrounding habitat.

Defining both *w* and $\phi$ as log-linear models allows movement and habitat covariates to be combined into a single selection function (termed an “integrated” SSF; [2]). That is, the SSF takes the form,

2 $$\begin{aligned} p(\varvec{y}_{t+1} \mid \varvec{y}_t) = \frac{\exp \{\varvec{c}_h(\varvec{y}_t, \varvec{y}_{t+1}) \cdot \varvec{\beta }_h\} \exp \{\varvec{c}_m(\varvec{y}_t, \varvec{y}_{t+1}) \cdot \varvec{\beta }_m\}}{\int _{\varvec{z}\in \Omega } \exp \{\varvec{c}_h(\varvec{y}_t, \varvec{z}) \cdot \varvec{\beta }_h\} \exp \{\varvec{c}_m(\varvec{y}_t, \varvec{z})\cdot \varvec{\beta }_m\}d\varvec{z}} \end{aligned}$$

where $\varvec{c}_h(\varvec{y}_t, \varvec{y}_{t+1})$ and $\varvec{c}_m(\varvec{y}_t, \varvec{y}_{t+1})$ are vectors of habitat and movement covariates (respectively), $\varvec{\beta }_h$ and $\varvec{\beta }_m$ are the associated vectors of selection coefficients, and $\cdot$ is the dot product. Through factorisation, this can be simplified to

3 $$\begin{aligned} p(\varvec{y}_{t+1} \mid \varvec{y}_t) = \frac{\exp \{\varvec{c}(\varvec{y}_t, \varvec{y}_{t+1}) \cdot \varvec{\beta }\}}{\int _{\varvec{z}\in \Omega } \exp \{\varvec{c} (\varvec{y}_t, \varvec{z}) \cdot \varvec{\beta }\}d\varvec{z}} \end{aligned}$$

where

4 $$\begin{aligned} \varvec{c}(\varvec{y}_t, \varvec{y}_{t+1}) = \begin{pmatrix} \varvec{c}_h(\varvec{y}_t, \varvec{y}_{t+1}) \\ \varvec{c}_m(\varvec{y}_t, \varvec{y}_{t+1}) \end{pmatrix} \end{aligned}$$

and $\varvec{\beta } = (\varvec{\beta }_h, \varvec{\beta }_m)$. This formulation makes the dependence between movement and habitat selection clear, and explicitly allows interactions between the two [2]. The SSF parameters for habitat covariates indicate the animal’s preference (i.e., positive coefficient) or avoidance (i.e., negative coefficients) of environmental features. For a coefficient $\beta$, $\exp (\beta )$ is the multiplicative effect to the SSF of an increase of one unit in the corresponding covariate, all else being constant. This quantity is called the relative selection strength (RSS; [1]), and is commonly used to interpret habitat selection models. Habitat covariates can be any spatial feature that affects how animals move and use space. There is a wide range of potential covariates, such as foraging resources (e.g., vegetation type, prey density), features that affect the ease of movement (e.g., forest cover, elevation), or proxies of risk (e.g., distance to roads, predator density).

SSFs can be parametrised in such a way that the coefficients for movement covariates represent the parameters of step length and turning angle distributions. It has been shown that the SSF parameter associated with the cosine of the turning angle (denoted $\beta _\theta$) is an unbiased estimator of the concentration parameter of a von Mises distribution [2, 9], with a mean of 0 or $\pi$ (depending on the sign of $\beta _\theta$; full details in Additional file 1: Appendix A). That is, $\beta _\theta$ can be translated to the mean $\mu _\theta$ and angular concentration $\kappa$ of the von Mises distribution as,

5 $$\begin{aligned} \mu _\theta = {\left\{ \begin{array}{ll} 0 &{} \text {if } \ \beta _\theta \ge 0 \\ \pi &{} \text {if } \ \beta _\theta < 0 \end{array}\right. }, \quad \text {and } \kappa = |\beta _\theta |. \end{aligned}$$

In this formulation, the mean of the turning angle distribution can only be zero (directional persistence) or $\pi$ (reversion in direction); in practice, this is not very limiting because these are the most common scenarios found in animal movement data [28].

Similarly, step lengths can be modelled with various distributions in the exponential family, via the inclusion of specific covariates in the SSF. The general idea is to take the probability density function of $\varvec{y}_{t+1} \mid \varvec{y}_t$ that would arise from a given distribution of step lengths, and write it in the form of Eq. 3 to identify the relevant covariates $\varvec{c}_m$. In this paper, we focus on implementing a gamma distribution of step lengths, but many other distributions could be used. For example, Forester et al. [14] shows how to model steps with a Weibull distribution, and Avgar et al. [2] extends this to other exponential family members (e.g., a half-normal distribution can accomodate step lengths of zero). Additional file 1: Appendix A, and Avgar et al. [2] derive the suitable covariates, and it is shown that step length and its log can be used to model a gamma distribution of step lengths. The associated coefficients, $\beta _L$ and $\beta _{\log (L)}$, are related to the mean $\mu _L$ and standard deviation $\sigma _L$ of the gamma distribution through

6 $$\begin{aligned} \mu _L = \frac{\beta _{\log (L)} + 2 }{\beta _L},\quad \text {and } \sigma _L = - \frac{\sqrt{\beta _{\log (L)} + 2}}{\beta _L}. \end{aligned}$$

Note that the parameters of a gamma distribution are stricly positive, whereas the $\varvec{\beta }_m$ coefficients are typically estimated with no constraints. As a result, the estimated parameters may sometimes not correspond to a valid step length distribution; in such cases, it may be necessary to use a different distribution, or to constrain the parameters during model fitting.

### State-switching SSFs

Following Nicosia et al. [30], we formulate a state-switching version of an SSF using HMMs. HMMs are doubly stochastic models, in which observation variables $\{\varvec{Y}_1, \varvec{Y}_2, \dots , \varvec{Y}_T\}$ arise from state-dependent distributions determined at each time $t \in \{1, 2, \dots , T\}$ by the latent state variable $S_t \in \{1, 2, \dots , K\}$ [50]. The number of states *K* is not estimated, but is chosen based on expert knowledge, research questions, and model checking (see [33], for guidance on the number of states). The estimated states are typically considered to represent behavioural states of the animal (e.g., encamped, foraging, travelling), but it is up to the practitioner to draw these inferences based on the estimated state parameters [28, 29]. A basic, first-order HMM assumes that the hidden states are a Markov chain, where the state at time *t* is dependent only on the previous state at time $t-1$. This state process is characterised by the transition probabilities, given as a $K \times K$ matrix

7 $$\begin{aligned} \varvec{\Gamma } = \begin{pmatrix} \gamma _{11} &{} \cdots &{} \gamma _{1K} \\ \vdots &{} \ddots &{} \vdots \\ \gamma _{K1} &{} \cdots &{} \gamma _{KK} \end{pmatrix} \end{aligned}$$

where $\gamma _{ij} = \text {Pr}(S_{t+1} = j \mid S_t = i)$ is the probability of switching from state *i* to state *j* over one time interval [19]. The observation model describes how the data are related to the hidden states. We denote the density of observation $\varvec{y}_t$ in state *k* as $p_k(\varvec{y}_t) = p(\varvec{Y}_t =\varvec{y}_t \mid S_t = k)$.

The HMM-SSF is a special case of an HMM, where the distributions $p_k$ are given as SSFs with state-specific parameters. At each time *t*, an animal selects a step according to one of *K* SSFs, as determined by the state at *t*, where each SSF has its own set of selection coefficients (defining movement patterns and habitat preferences in that state). Therefore, the density of a step ending at $\varvec{y}_{t+1}$ given that it is in state *k* and started at $\varvec{y}_t$ is given by the following log-linear SSF,

8 $$\begin{aligned} p(\varvec{y}_{t+1} \mid S_t = k, \varvec{y}_t) =\frac{\exp \{\varvec{c}(\varvec{y}_t, \varvec{y}_{t+1}) \cdot \varvec{\beta }^{(k)}\}}{\int _{\varvec{z}\in \Omega } \exp \{\varvec{c}(\varvec{y}_t, \varvec{z}) \cdot \varvec{\beta }^{(k)} \}d\varvec{z}} \end{aligned}$$

where $\varvec{c}$ is a vector of covariates and $\varvec{\beta }^{(k)}$ are the associated selection coefficients in state *k*. The likelihood is also conditional on $\varvec{y}_{t-1}$ if turning angle is included, as three successive locations are required for its calculation. Throughout the paper, we focus on the exponential SSF, as this is most common and allows flexible movement-habitat interactions via the selection function (e.g., state-specific movement speed may depend on habitat features; [2]). An interaction term within the state-specific SSF can be viewed analogously to including covariates on the observation parameters (e.g., step length mean) of a standard HMM. However, note that the state-specific SSFs do not have to follow Eq. 8, and can take any valid SSF form (e.g., with flexible distributions of $\phi$ and *w*; [14]).

The HMM-SSF relaxes the usual assumption of SSF models that successive steps are independent, by inducing dependence through the state process. That is, although standard SSFs capture some dependence between locations through movement covariates, the HMM-SSF explicitly models additional dependence between successive steps through the Markov chain. This can capture temporal correlation in movement and habitat selection patterns. For example, high probabilities on the diagonal of the transition matrix might imply that a step with high selection for a particular resource is likely to be followed by another step with a similar level of selection (and likewise for movement correlation). Therefore, the HMM-SSF improves inferences from using step selection analysis on its own, where selection is averaged over all steps. In the rest of this paper, we describe how well-known algorithms and extensions developed for HMMs and SSFs can be adapted to the present context.

#### Time-varying transition probabilities

We extend the model of Nicosia et al. [30] to include covariates on the transition probabilities. At time *t*, each transition probability is linked to covariates using a multinomial logit link

9 $$\begin{aligned} \gamma _{ij}^{(t)} = \text {Pr}(S_{t+1} = j \mid S_t = i) =\frac{\exp (\eta _{ij}^{(t)})}{\sum _{k=1}^{K}\exp (\eta _{ik}^{(t)})} \end{aligned}$$

with the linear predictor for *P* covariates $\{\omega _1^{(t)}, \omega _2^{(t)}, \dots , \omega _P^{(t)}\}$ given as

10 $$\begin{aligned} \eta _{ij}^{(t)} = {\left\{ \begin{array}{ll} \alpha ^{(ij)}_0 + \sum _{p=1}^{P} \alpha _p^{(ij)} \omega _p^{(t)} &{} \text {if } i \ne j \\ 0 &{} \text {otherwise.} \end{array}\right. } \end{aligned}$$

For each transition probability, $\alpha _0^{(ij)}$ is an intercept parameter, and $\alpha _p^{(ij)}$ measures the effect of the *p*-th covariate $\omega _p$ [28]. Note that transition probability covariates will be different to those included in the SSF. Generally, SSF covariates are spatial features that affect step-level movement decisions, whereas covariates included on the transition probabilities are expected to determine the probability of moving into each behavioural state. Among others, transition probability covariates could be temporal variables (e.g., time of day, season), individual-level attributes (e.g., body condition, sex, age), or habitat features (Patterson et al. 2009, Langrock et al. 2012). Transition probability covariates are not “selected” or “avoided” by animals, but they may affect habitat selection behaviour, and this is captured by Eq. 10.

In some cases, there might be good reasons to include a covariate either in the SSF (i.e., as $\varvec{c}(\varvec{y}_t, \varvec{y}_{t+1})$ in Eq. 6), or on the transition probabilities (i.e., as $w_p^{(t)}$ in Eq. 8), which would lead to different interpretations. For example, a food resource could be perceived as triggering an animal’s transition into a foraging behavioural state, or the foraging behaviour could be defined as selection for that food resource. Although the covariate could in principle be included in both model components, this might cause estimation problems if the two effects cannot be adequately separated. We suggest using biological knowledge to decide where in the model to include the covariate, and note that this will likely affect the interpretation of the states.

Recently, Prima et al. [37] also modelled the transition probability of an HMM-SSF as a function of covariates, but did so in a two-stage approach. They defined a binary response based on the probability that the animal transitioned at each time step, which was modelled with a binomial generalised linear model with an environmental predictor. This two-stage approach is not uncommon in HMM analyses (e.g., [6]), but it can be problematic because it does not account for the uncertainty in state classification and it does not allow the state categorisation to depend on environmental covariates. Therefore, direct inclusion of covariates on the transition probabilities is preferable, and relatively straightforward using standard HMM approaches [19, 21, 31].

### Implementation

There are two main components to the implementation of the HMM-SSF: (i) approximation of the SSF likelihood, and (ii) maximum likelihood estimation of the HMM-SSF parameters via the forward algorithm. In the next two sections, we compute approximate state-specific SSF likelihoods for all steps based on numerical integration, and these are used as the state-dependent densities in the HMM likelihood. All implementation code is accessible in the Additional file 1.

#### Integration in the SSF likelihood

In practice, the integral in the denominator of Eq. 8 is analytically intractable [39]. However, we can use Monte Carlo integration (i.e., evaluating the function at random points) to get an approximation of the likelihood [17, 35]. In SSFs, this is often referred to as a “case–control” design, in which random locations (i.e., the controls) are matched spatially and temporally to each observed location (i.e., the case) to approximate each step likelihood. For each time step *t*, we sample *N* control locations $\{\varvec{z}_{1t}, \varvec{z}_{2t}, \dots , \varvec{z}_{Nt} \}$ and the state-dependent density is approximated as,

11 $$\begin{aligned} \tilde{p}(\varvec{y}_{t+1} \mid S_t = k, \varvec{y}_t) =\frac{\exp \{\varvec{c}(\varvec{y}_t, \varvec{y}_{t+1}) \cdot \varvec{\beta }^{(k)}\}}{\frac{1}{N+1} \sum _{i = 0}^N\exp \{\varvec{c}(\varvec{y}_t, \varvec{z}_{it}) \cdot \varvec{\beta }^{(k)} \}} \end{aligned}$$

with $\varvec{z}_{0t} = \varvec{y}_{t+1}$. The term $1/(N+1)$ in the denominator is often omitted when it is constant; here, we include it explicitly as the number of valid control locations may vary between steps due to missing covariate data. In general, the choice of *N* will impact how well the function is approximated, where as $N \rightarrow \infty$ the approximation approaches the true likelihood.

The method to generate control locations in an SSF is an important choice, with potential implications on the precision of the estimation. Equation 11 requires that controls be sampled uniformly over $\Omega$. In practice, it is more common to simulate control locations on a disc that is sufficiently large to encompass the vast majority of the probability mass, as increasing radius size much beyond the maximum observed step does little to improve the estimation [18]. However, uniform sampling can require a large number of random locations to achieve low error, as animals are generally unlikely to take long step lengths and many of the sample controls will have a likelihood close to zero. One way to increase computational efficiency and the precision of the approximation is to preferentially sample where the likelihood is expected to be highest, using importance sampling. If the random locations $\varvec{z}_{it}$ are generated from a distribution with probability density function *h* (which typically also depends on the previous observed location $\varvec{y}_t$), we can write the approximate likelihood as,

12 $$\begin{aligned} \tilde{p}(\varvec{y}_{t+1} \mid S_t = k, \varvec{y}_t) =\frac{\exp \{\varvec{c}(\varvec{y}_t, \varvec{y}_{t+1}) \cdot \varvec{\beta }^{(k)}\}}{\frac{1}{N+1} \sum _{i=0}^N \exp \{\varvec{c}(\varvec{y}_t, \varvec{z}_{it}) \cdot \varvec{\beta }^{(k)} \} / h(\varvec{y}_t, \varvec{z}_{it} )} \end{aligned}$$

where $z_{0t} = \varvec{y}_{t+1}$. For example, random locations could be generated based on gamma-distributed distances from $\varvec{y}_t$, to take advantage of the animal’s tendency to favour short steps, and *h* would be the corresponding two-dimensional spatial distribution (Additional file 1: Equation 2 of Appendix A.1).

#### Direct likelihood maximisation via the forward algorithm

We evaluate the likelihood of the HMM-SSF using a recursive algorithm (i.e., the forward algorithm), which efficiently accounts for all possible state sequences based on the dependence structure of the model [19, 28]. The model likelihood can be written as

13 $$\begin{aligned} L(\varvec{y}_1, \dots , \varvec{y}_T \mid \varvec{\beta }, \varvec{\alpha }) =\varvec{\delta } \varvec{P}(\varvec{y}_1, \varvec{y}_2) \varvec{\Gamma }^{(2)} \varvec{P}(\varvec{y}_2, \varvec{y}_3) \cdots \varvec{\Gamma }^{(T-1)} \varvec{P}(\varvec{y}_{T-1}, \varvec{y}_T) \textbf{1}' \end{aligned}$$

where $\varvec{\delta } = (\Pr (S_1 = 1), \dots , \Pr (S_1 = K))$ is the initial distribution of the state process, $\varvec{P}(\varvec{y}_t, \varvec{y}_{t+1})$ is a diagonal matrix with *k*-th element given as the approximate transition density $\tilde{p}(\varvec{y}_{t+1} \mid S_t = k, \varvec{y}_t)$ (obtained via Monte Carlo integration or importance sampling; Eq. 11 or 12), $\varvec{\beta }$ is a vector of the SSF parameters, $\varvec{\alpha }$ is a vector of the transition probability parameters, and $\mathbf {1'}$ is a column vector of ones. If there are missing observations, we account for these by defining the corresponding $\varvec{P}$ as the identity matrix [19]. The initial distribution $\varvec{\delta }$ is often susceptible to identifiability issues and, to avoid numerical problems in the estimation, we fix it to be the stationary distribution of the transition probability matrix at time $t = 1$ [28]. The Markov chain is not stationary if there are covariates included on the transition probabilities but, even in that case, we assume that the stationary distribution of $\varvec{\Gamma }^{(1)}$ is a good heuristic choice for the initial distribution. To prevent underflow, we implement the forward algorithm for the “scaled” negative log-likelihood of the HMM-SSF, following Zucchini et al. [50]. In the case of multiple individuals in the same data set, we assume that all individuals share the same parameters (“complete pooling”; [19, 28]).

To obtain estimates of all parameters, the negative log-likelihood of the model can then be minimised with respect to $\varvec{\alpha }$ and $\varvec{\beta }$ using a numerical optimiser, and we use optim in R. This approach stands in contrast with the expectation-maximisation (EM) algorithm proposed by Nicosia et al. [30] to fit the HMM-SSF. Although the two methods will generally converge to the same estimates, it has been argued that direct likelihood maximisation is often faster and easier to implement for HMMs [21, 22, 50]. Parameter standard errors can then be computed as the square root of the diagonal elements of the inverse Hessian matrix [50]. For covariate-dependent transition probabilities, confidence intervals are obtained using the delta method [49]. Reliable optimization in HMMs can be sensitive to choice of initial parameter values [28]. A common solution is to generate many sets of initial values, use each to fit the model, and finally keep the solution with the lowest negative log-likelihood (implemented in Sect. 2.4). Another approach could be to use the two-stage approach (HMM then SSF, e.g., [32, 41]) to find reasonable starting values, and this could be particularly important for complex model formulations or to aid in variable selection. However, this preliminary model exploration would assume that the states are largely defined by movement, and although this is likely in many cases, it should be used cautiously. We used simulations to check that our model fitting procedure can recover all model parameters when the true data generating process is known. Full details are given in Additional file 1: Appendix B.

#### State decoding

In many situations, it is of interest to estimate the state process $S_t$, a procedure called state decoding. We expect that state decoding will be relevant to most HMM-SSF analyses as a method to identify behavioural phases from movement data. The two main approaches to tackle this problem for HMMs are global and local decoding [50]. Global decoding consists of identifying the state sequence that is most likely to have given rise to the observed data, and can be computed using an efficient iterative algorithm (“Viterbi algorithm”; [50]). The output is a sequence of state indices, which can be used to subset or spatially visualise the locations by state. Alternatively, local decoding provides probabilities of occupying each state at each observation time (i.e., the state probabilities), which is often more informative about the uncertainty in state classification (where values close to 0.5 indicates high uncertainty in the state process). Here, we suggest computing the local state probabilities with the forward-backward algorithm [50]. Note that this is equivalent to the approach described by Nicosia et al. [30] to obtain the state probabilities as a by-product of their expectation-maximisation algorithm. A state sequence can be obtained from local state probabilities, by taking the state with highest probability at each time step. Although this is usually very similar to the Viterbi sequence, it is generally not identical, because they solve different optimisation problems (i.e., global decoding optimises over the full state sequence, rather than at each time step; [50]).

#### Simulating space use

A proposed application of SSFs is to simulate from the fitted model to estimate the utilisation distribution of the animal [42], and a similar method can be used for the HMM-SSF. This can give information about areas that are important for different behaviours, which is typically not possible with standard HMM approaches [16]. There are several ways to simulate utilisation distributions, which range from generating a single long track (i.e., the steady-state distribution) or several shorter tracks (i.e., a transient distribution; [42]). In this paper, we do not intend to provide guidelines for best simulation practice, but we present an algorithm to simulate data from the HMM-SSF (Additional file 1: Appendix B), which can then be used to estimate utilisation distributions. The general steps of the algorithm are to first simulate a state sequence based on the estimated transition probabilities, and then for each time step, simulate the next location from the SSF corresponding to the current state. In practice, this is done by proposing many possible next locations (within a disc), and selecting with probability proportional to their state-specific SSF (Eq. 9). Note that the number of proposed end points depends on the size of the disc, and it should be high enough to ensure good sampling coverage, so that bias is not introduced through the simulation. As implemented in Forester et al. [14], it would also be possible to simulate endpoints from an importance distribution *h*, which may be more computationally efficient. This would require a correction step (i.e., to re-weight the endpoints by their importance density) within the simulation procedure. In either case, the utilisation distribution can then be estimated using a method such as kernel density estimation on the simulated locations. This can be either an overall distribution based on all locations, or a behaviour-specific distribution if only locations in a given state are kept. The simulations should be designed to best capture the study system, and we provide one example in the next section, with more specific parameters and settings.

### Illustrative example

**Fig. 1:**
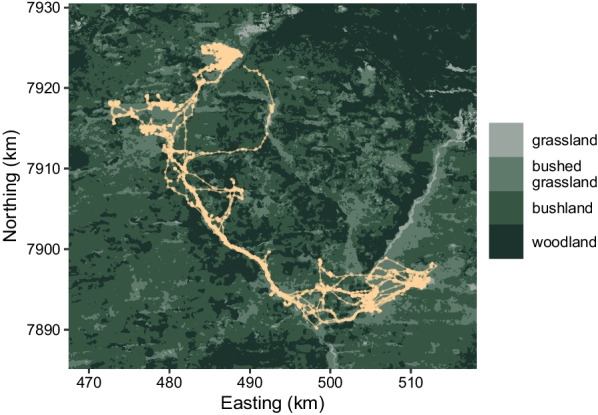
Habitat type map and zebra track (tan). Plot adapted from Michelot et al. [27]

We provide an example to demonstrate the workflow for implementing and interpreting the HMM-SSF. We analysed a track of plains zebra locations collected at a 30-minute resolution from January - April 2014 in Hwange National Park in Zimbabwe [27]. The time-series consisted of 7246 observations, with 125 missing locations. We fitted a two-state HMM-SSF, where the SSF component captured selection for a combination of habitat and movement covariates, and the HMM component captured behaviour change as a function of time of day. Two states are commonly used in movement analyses, which typically correspond to slow (i.e., encamped) and fast (i.e., exploratory) behavioural states (which have been previously identified in zebras; [37]). We included a categorical covariate for vegetation type, with four levels: grassland (reference category), bushed grassland, bushland, and woodland (Fig. 1). We modelled step lengths with a gamma distribution (i.e., with step and its log as covariates), and turning angle with a von Mises distribution (i.e., with the cosine of turning angle as a covariate). For transition probabilities, we included time of day $\tau$ as a cyclic covariate (following [48]), such that the linear predictor in Eq. 10 becomes

14 $$\begin{aligned} \eta _{ij}^{(t)} = {\left\{ \begin{array}{ll} \alpha ^{(ij)}_0 + \alpha _1^{(ij)} \cos \left( \frac{2\pi \tau _t}{24}\right) +\alpha _2^{(ij)} \sin \left( \frac{2 \pi \tau _t}{24}\right) &{} \text {if } i \ne j \\ 0 &{} \text {otherwise} \end{array}\right. } \end{aligned}$$

for $i, j \in \{1, 2\}$. All fitting was done with the implementation methods described in Sect. 2.3. To approximate the integral (via importance sampling), we generated $N = 25$ control locations for each case location. Control step lengths were generated as random draws from a gamma distribution with the mean and standard deviation of the observed data, and turning angles were generated as random draws from a uniform distribution. To ensure convergence of the estimation procedure, we fitted the model with several sets of initial values, spanning different patterns of selection, and chose the model with the lowest negative log likelihood. Lastly, we derived the relative selection strength (RSS) of grassland (i.e., reference category) compared to habitat type *j* as $1/\exp (\beta _j)$, where $\beta _j$ is the selection coefficient for habitat type *j*. The RSS for grassland can be interpreted as how much more likely a zebra is to take a step in grassland, compared to the other habitat type.

We determined the most likely sequence of states using the Viterbi algorithm, and the state probabilities at each time step using the forward-backward algorithm [28, 50]. In addition to the transition probabilities, we derived stationary state probabilities as functions of the time of day. For a given time of day, these provide some indication of the probability of being in each state, which is often useful for interpretation [31]. In practice, they are derived as the stationary distribution of the transition probability matrix for each time of day. Based on the fitted model, we also simulated a utilisation distribution. We generated five long tracks of 100,000 locations at a 30-minute interval. First, the state sequence was simulated based on the time of day, which was initialised as the first observed time. Then, each location was selected (with probabilities given by their SSF values) from a possible 10,000 endpoints on a disc with the radius $r = \text {max}(L) \times 1.1$, where $\text {max}(L)$ is the maximum observed step length. We chose to run five tracks (rather than one long track), so that we could run each simulation in parallel. The starting location of each track was chosen randomly from the observed data, and we removed the first 1000 locations of each track to reduce the effect of this choice. We used a reflective boundary condition, in which each simulated track was constrained to stay within the study area by fixing the SSF of any point outside the boundaries to zero. We then estimated the utilisation distribution by applying kernel density estimation (bandwidth = 2) to the simulated locations.

## Results

The simulation results (in Additional file 1: Appendix B) suggested that all components of the model were generally estimated well, including step length, turning angle, and habitat selection parameters, as well as transition probabilities as functions of a covariate. However, one habitat selection parameter was estimated poorly, which indicated that bias can arise when the spatial scale of covariate autocorrelation is much larger than the scale of movement.

**Fig. 2:**
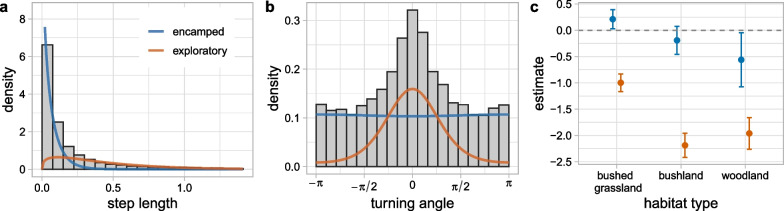
Movement and habitat selection estimates in zebra analysis. Estimated **a** step length and **b** turning angle distributions, weighted by the number of observations predicted to be in each state. The histograms show the empirical distributions of the data. The step length (x-axis) was truncated to the 99th percentile for visualisation purposes. **c** Habitat type parameter estimates (grassland is the reference category, i.e., corresponding to zero)

In the zebra analysis, we identified two states with distinct movement and habitat selection patterns. Table 1 provides a full list of selection parameter estimates with uncertainty, but here, we discuss these in terms of the parameters of the state-specific step length distribution (gamma with mean $\mu _L^{(k)}$ and standard deviation $\sigma ^{(k)}$; both in km) and von Mises distribution (angular concentration $\kappa ^{(k)}$ and mean $\mu ^{(k)}_{\theta } \in \{0, \pi \}$). State 1 was identified as a slow state ($\mu _L^{(1)} = 0.06$, $\sigma ^{(1)} = 0.07$) with no directional persistence ($\mu ^{(1)}_{\theta } = \pi$, $\kappa ^{(1)} = 0.01$). State 2 was characterised by faster movement ($\mu _L^{(2)} = 0.43$, $\sigma ^{(2)} = 0.37$) with higher directional persistence ($\mu ^{(2)}_{\theta } = 0$, $\kappa ^{(1)} = 1.46$) (Fig. 2a, b). We consider these to be encamped and exploratory behaviours, respectively [29].

**Table 1:** Estimated parameters from the HMM-SSF fitted to zebra telemetry data

	State/transition	Covariate		Estimate (95% CI)
Movement (SSF)	*Encamped*	Step length *L*	$\beta ^{(1)}_1$	$-13.6\ (-14.8,\ -12.3)$
		log(*L*)	$\beta ^{(1)}_2$	$-1.18\ (-1.21,\ -1.15)$
		cos($\theta$)	$\beta ^{(1)}_3$	$-0.01\ (-0.07,\ 0.04)$
	*Exploratory*	Step length *L*	$\beta ^{(2)}_1$	$-3.11\ (-3.36,\ -2.86)$
		log(*L*)	$\beta ^{(2)}_2$	$-0.65\ (-0.78,\ -0.53)$
		cos($\theta$)	$\beta ^{(2)}_3$	$1.46\ (1.33,\ 1.59)$
Habitat (SSF)	*Encamped*	Bushed grassland	$\beta ^{(1)}_4$	$0.21\ (0.03,\ 0.39)$
		Bushland	$\beta ^{(1)}_5$	$-0.19\ (-0.46,\ 0.08)$
		Woodland	$\beta ^{(1)}_6$	$-0.56\ (-1.08,\ -0.05)$
	*Exploratory*	Bushed grassland	$\beta ^{(2)}_4$	$-1.00\ (-1.16,\ -0.83)$
		Bushland	$\beta ^{(2)}_5$	$-2.19\ (-2.42,\ -1.96)$
		Woodland	$\beta ^{(2)}_6$	$-1.96\ (-2.26,\ -1.66)$
Transition probabilities (HMM)	*Encamped − exploratory*	Intercept	$\alpha _0^{(1,2)}$	$-1.83\ (-1.98,\ -1.69)$
		cos$\left( \frac{2 \pi \tau }{24}\right)$	$\alpha _1^{(1,2)}$	$0.03\ (-0.14,\ 0.21)$
		sin$\left( \frac{2 \pi \tau }{24}\right)$	$\alpha _2^{(1,2)}$	$0.81\ (0.63,\ 0.99)$
	*Exploratory − encamped*	Intercept	$\alpha _0^{(2,1)}$	$-1.26\ (-1.40,\ -1.12)$
		cos$\left( \frac{2 \pi \tau }{24}\right)$	$\alpha _1^{(2,1)}$	$0.15\ (-0.02,\ 0.33)$
		sin$\left( \frac{2 \pi \tau }{24}\right)$	$\alpha _2^{(2,1)}$	$0.14\ (-0.04,\ 0.32)$

Note, these are the untransformed $\beta$ estimates and do not directly represent the mean and variance of the assumed gamma distribution; the turning angle (given by $\theta$) parameter represents the angular concentration of the von Mises distribution. *L* is the step length (km) and $\tau$ is the hour of the day.

Habitat selection patterns varied between the two states, although both showed generally high selection for the habitat types with higher grassland cover (Fig. 2c). In the encamped state, the zebra selected for grassland over all habitat types, except for bushed grassland, which had a positive selection coefficient. Compared to bushed grassland, the grassland (i.e., reference) RSS was 0.8 (i.e., the zebra was 0.8 times as likely to select grassland than bushed grassland). When encamped, the zebra was more likely to choose grassland over bushland and woodland: the RSS was 1.2 compared to bushland, and 1.8 compared to woodland. Only bushland coefficient had a 95% CI that overlapped zero, but the uncertainty was generally high and the other CIs were also close to overlapping zero (Fig. 2c).

Habitat selection was stronger in the exploratory state, where there was clear avoidance of all habitat types relative to grassland and no CIs overlapped zero. The grassland RSS was 2.7, 8.9, and 7.1 for bushed grassland, bushland, and woodland (respectively). Positive selection for grassland is consistent with previous results from Michelot et al. [27], and may represent selection for their main foraging resources. However, neither state seems to fully capture foraging behaviour in this example, which may explain the selection for grassland or bushed grassland in both states. This may suggest that a 3-state model might be better able to distinguish between biological behaviours in this example [33].

**Fig. 3:**
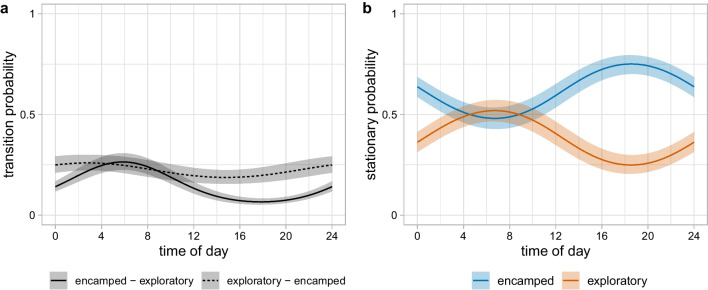
Effect of time of day on transition probabilities in zebra analysis. **a** Estimated transition probabilities $\gamma _{12}$ and $\gamma _{21}$ as functions of time of day. **b** Derived stationary state probabilities as functions of time of day. The shaded areas are 95% pointwise confidence bands

**Fig. 4:**
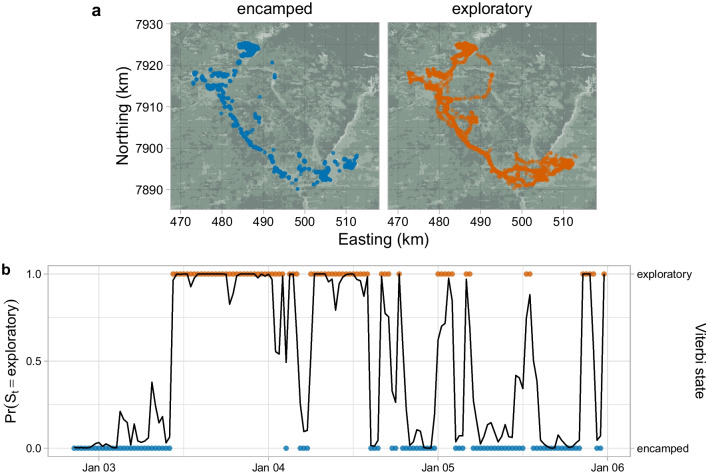
State decoding for zebra example, where blue points are encamped and orange points are exploratory. **a** Locations in each state, estimated via global decoding (Viterbi algorithm). **b** Estimated probability of being in the exploratory state (black line) and Viterbi sequence (coloured points) over a few days chosen for visualisation purposes (note separate y-axes)

We found an effect of time of day on the transition probabilities and on the stationary state probabilities (Fig. 3). There was an increase in the probability of transitioning into exploratory in the morning (highest at approximately 07:00), but no strong effect on the probability of transitioning from exploratory to encamped (Fig. 3a). The stationary probability of being in the encamped state was highest between 15:00 and 23:00 (peak at roughly 19:00), and lowest between 03:00 and 11:00 (trough at roughly 07:00; Fig. 3b). This suggests that this zebra was less active in the late afternoon and evening, and more active in the morning. Maps of the locations classified in each state by the Viterbi algorithm confirm that the encamped state tended to be localised and clustered, where the exploratory state was more spatially diffuse (Fig. 4a). The local state probabilities were in agreement with the Viterbi sequence, and the highest state probability was larger than 0.75 for about 85% of time steps (Fig. 4b).

**Fig. 5:**
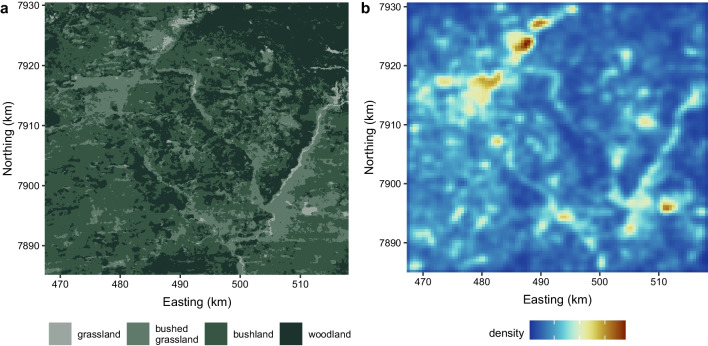
**a** Habitat type map used in the analysis. **b** Estimated utilisation distribution (where blue is lowest density and brown is highest), derived from five simulated tracks of 100,000 locations each. The tracks were simulated based on the estimated HMM-SSF parameters

We showed that it is possible to estimate large-scale space use from the HMM-SSF, via simulation (Fig. 5). The SSF parameter indicated that movement was driven by selection for grassland and bushed grassland, and this was clearly captured in the simulated utilisation distribution.

## Discussion

In this paper, we improved the flexibility and applied utility of the HMM-SSF, building on recent works by Nicosia et al. [30] and Prima et al. [37]. Depending on the aims and experience of the practitioner, the HMM-SSF can be viewed as a standard HMM with a habitat selection observation process, or as an SSF that allows for state-switching dynamics. Below, we further describe how the HMM-SSF extends these popular modelling frameworks, and discuss the associated implementation challenges and potential extensions.

### Combining HMMs and SSFs

Viewing this model as a standard HMM opens the way for a wide range of computational tools and extensions, including direct optimisation of the likelihood with the forward algorithm, local and global decoding of the state process, and covariate effects on the transition probabilities. Including additional observation variables in HMMs has been advocated as a method to identify biologically-relevant behavioural states [24], but most HMM analyses still focus solely on movement. In the HMM-SSF, the animals’ behaviour within each state is defined by both movement and habitat selection characteristics. This approach will be particularly effective when habitat variables are closely linked to behaviour (e.g., a foraging resource will help identify foraging behaviour), and when state transitions depend on temporal or individual-specific factors (e.g., behaviours occur seasonally, or vary by sex, age, etc.).

The HMM-SSF can also be viewed as an improvement over SSFs, via the inclusion of multiple behavioural states. The state-specific SSFs retain the flexibility of other SSF approaches, and can incorporate movement-habitat interactions and temporal effects [2, 40], and the parameter interpretation remains the same (e.g., relative selection strength, following [1]). The ability to separate behavioural states better accounts for temporal autocorrelation in the data, and can reveal nuanced patterns of habitat selection that would disappear in a standard SSF (e.g., an animal alternating between selection and avoidance of some spatial feature). Deriving space use from SSFs is a commonly desired application, as large-scale patterns of interest arise from small-scale movement decisions [36, 42]. Space use from the HMM-SSF considers non-homogeneous selection parameters, and should be an improvement on single-state SSFs. Although we focused on deriving an overall distribution, there are likely to be many cases where the state-specific distributions are of interest (i.e., when resource selection varies strongly between states), and these are straightforward to derive from the simulated data. We intend our simulation to be a simple illustration, and more refined stochastic and analytical methods could be explored for the HMM-SSF (e.g., methods to upscale from SSFs are reviewed in [36]).

Avgar et al. [2] showed how the exponential form of the SSF can be beneficial when estimating movement and habitat selection simultaneously (i.e., to include movement interactions and implement the SSF as conditional logisitic regression). However, this approach reduces flexibility in the possible movement distributions, as they must be from the exponential family and the angular mean of the von Mises distribution is constrained to be zero or $\pi$. There may be scenarios in which these assumptions are not realistic, and other distributions would be more suitable. For example, turning angles are sometimes better modelled by the wrapped Cauchy distribution (which cannot be written in the exponential form; [8, 28]), possibly with multimodality [4]. The HMM-SSF can be formulated to accommodate a wide range of movement distributions, by specifying the movement kernel $\phi$ of the SSF as any parametric distribution of step length and turning angles [14], or by including movement variables as separate data streams in the HMM. However, these approaches may preclude movement-habitat interactions, and the latter assumes that movement variables are independent of the SSF variables given the state (which may bias SSF parameters; [14]). Therefore, these drawbacks need to be weighed against the benefits of more flexible movement modelling in each study-specific context.

### Implementation challenges

The model we presented inherits some of the limitations of SSFs and HMMs, such as scale dependence and implementation challenges. The HMM-SSF is formulated in discrete time, where the estimated parameters are scale-dependent and only describe movement, behaviour, and habitat selection at the time interval of the observed movement step (a known problem in movement ecology: [13, 26]). Additionally, if the SSF is implemented with Monte Carlo integration, it is important to consider how the sampling scheme may affect the precision of the estimation. To improve the approximation, we suggest using importance sampling based on observed step length distribution. State-specific Monte Carlo sampling may be more suitable in cases where the states have very different movement characteristics, but this would likely be very computationally intensive. Future work could explore how the inferences and performance of the HMM-SSF are affected by the chosen method of integration and scale of observations.

The flexibility of the HMM-SSF requires a large number of parameters. Therefore, inference may be more numerically unstable and computationally costly than standard SSFs or two-stage approaches (e.g., [2, 32]). In particular, the HMM-SSF cannot be rewritten as a special case of conditional logistic regression (CLR), and model fitting requires implementing a custom likelihood function based on the forward algorithm (or integrating a weighted CLR routine within the EM algorithm, as in [30]). This increases computational cost compared to fast CLR software typically used for step selection analysis (e.g., clogit in the package survival; [45]). Further, although direct numerical likelihood optimisation is general to a broad range of model formulations, numerical stability might depend on model and landscape complexity. Models with many states, spatially autocorrelated habitats, or complex covariate interactions may be more difficult to fit. In such cases, practitioners may consider simulating data from the fitted model to assess numerical stability across multiple model fits. Simulations could also be used for model checking, where summary metrics of simulated data could be compared to the real data in an approach similar to those proposed for SSFs [12] and HMMs [25, 29]. Developing proper model checking techniques for the HMM-SSF will be important, and we note that a large sample of locations may be needed to ensure enough information is available from the data to reliably identify latent behavioural states.

### Conclusion

As data sets become larger and more varied with technological innovation, we expect the utility of the HMM-SSF to keep increasing. There are many possible extensions to this framework to study complex ecological phenomena, often with minimal changes to the implementation. The flexible HMM framework could be used to incorporate extensions, such as higher-order dependence (i.e., memory dynamics; [19]), feedback mechanisms [19, 20], and additional data streams (such as physiological or accelerometer data; [21]). Additional information about the state or observation process could be incorporated by formulating the model as a semi-supervised HMM (i.e., where states are known a priori; [21]). This framework could also be used to add a behavioural component in more general SSF formulations, including recent work on memory [46] and movement energetics [10, 18]. Therefore, a state-switching habitat selection model has many purposes in ecological research, while remaining accessible and interpretable.

## Supplementary information

**Additional file 1.** Appendix A explains the derivation of movement covariates for a gamma distribution of steps and a von Mises distribution of turning angles. Appendix B contains a simulation study to verify the implementation methods presented in the main text of the manuscript. All code is available at Zenodo and GitHub.

## Data Availability

The data and code to reproduce all analyses are archived on Zenodo (https://doi.org/10.5281/zenodo.7872602). A working version of the implementation code and further documentation is also available on GitHub at https://github.com/NJKlappstein/HMM-SSF.
