# Supplementary material for: Flexible hidden Markov models for behaviour-dependent habitat selection
Source: Mov Ecol. 2023 Jun 3;11:30. doi: 10.1186/s40462-023-00392-3 (PMC10239607; doi:10.1186/s40462-023-00392-3)
Supplement: Supplementary file 1 — Additional file 1. Appendix A explains the derivation of movement covariates for a gamma distribution of steps and a von Mises distribution of turning angles. Appendix B contains a simulation study to verify the implementation methods presented in the main text of the manuscript. All code is available at Zenodo and GitHub. [file 40462_2023_392_MOESM1_ESM.pdf]

A SSF details

A.1 Step length distribution

Here, we derive the SSF covariates needed to model step lengths with a gamma distribution. Following Rhodes et al. (2005) and Forester et al. (2009), the two-dimensional spatial distribution of the endpoint $\boldsymbol{y}$ of a step (given that it started at $\boldsymbol{x}$) is related to the distribution of the step length $L = ||\boldsymbol{y} - \boldsymbol{x}||$ as follows

$$p(\boldsymbol{y} \mid \boldsymbol{x}) = \frac{p(L)}{2\pi L}. \tag{1}$$

This defines the relationship between the two-dimensional distribution $p(\boldsymbol{y} \mid \boldsymbol{x})$ and the one-dimensional distribution of the distance L between the start point $\boldsymbol{x}$ and end point $\boldsymbol{y}$. Therefore, the distribution of $\boldsymbol{y}$ for gamma-distributed step lengths with shape a and scale b is obtained by replacing $p(L)$ by the gamma probability density function,

$$\phi(\boldsymbol{y} \mid \boldsymbol{x}) = p(\boldsymbol{y} \mid \boldsymbol{x}, a, b) = \frac{L^{a-1}\exp(-L/b)}{b^a\Gamma(a)} \times \frac{1}{2\pi L} \tag{2}$$

where Γ is the gamma function. To model this in the SSF, we write the movement kernel as the exponential of a linear predictor, such that $\phi(\boldsymbol{y} \mid \boldsymbol{x}) = \exp(\boldsymbol{c}(\boldsymbol{x}, \boldsymbol{y}) \cdot \boldsymbol{\beta})$. Therefore, to identify the terms of linear predictor, we can take the natural log of the movement kernel,

$$\log(p(\boldsymbol{y} \mid \boldsymbol{x}, a, b)) = \left(-\frac{1}{b}\right) L + (a-2)\log(L) - \log(b^a\Gamma(a)2\pi). \tag{3}$$

Note that the last term, $\log(b^a\Gamma(a)2\pi)$, is a constant (i.e., not dependent on the step length) and can be ignored. Finally, this leaves two terms that need to be included in the linear predictor of the SSF: step length L and its logarithm $\log(L)$. The equation gives us the relationship between the gamma distribution parameters and the selection coefficients (here, referred to as β_1 and β_2 for L and $\log(L)$, respectively): $\beta_1 = -1/b$ and $\beta_2 = a - 2$. In turn, we can derive mean μ and standard deviation σ of the step length distribution, in terms of the selection parameters. For the gamma distribution, it is known that

$$a = \frac{\mu^2}{\sigma^2} \quad \text{and} \quad b = \frac{\sigma^2}{\mu} \tag{4}$$

from which we can derive the following

$$\mu = -\frac{\beta_2 + 2}{\beta_1} \quad \text{and} \quad \sigma = -\frac{\sqrt{\beta_2 + 2}}{\beta_1} \ . \tag{5}$$

Here, we describe the approach for for the gamma distribution, but this could be applied to other distributions from the exponential family suitable to model step lengths (Forester et al., 2009; Avgar et al., 2016). For example, the exponential distribution is a special case of the gamma distribution, where $a = 1$.

A.2 Turning angle distribution

Similarly to the procedure used above for step length, we can derive covariates required to model a given distribution of turning angle. Following Avgar et al. (2016) and Nicosia et al. (2017), we focus our attention on the von Mises distribution, with probability density function

$$p(\theta \mid \mu, \kappa) = \frac{\exp(\kappa \cos(\theta - \mu))}{2\pi I_0(\kappa)}, \tag{6}$$

where $\theta \in (-\pi, \pi]$ is the turning angle, $\mu \in (-\pi, \pi]$ is the mean parameter, $\kappa > 0$ is the concentration parameter, and I_0 is the modified Bessel function of the first kind of order 0. Intuitively, κ is inversely related to the variance of the distribution, and large κ corresponds to a high peak around μ.

We first focus on the case where $\mu = 0$, corresponding to a tendency to persist in direction, which is the most prevalent in the context of animal movement (but we discuss the case $\mu = \pi$ below). Assuming $\mu = 0$, we take the log of the density function to identify terms that should be included in the SSF,

$$\log[p(\theta \mid 0, \kappa)] = \kappa \cos(\theta) - \log[2\pi I_0(\kappa)]. \tag{7}$$

The term $\log[2\pi I_0(\kappa)]$ does not depend on turning angle, and we can therefore omit it. Finally, we get the result that $\kappa \cos(\theta)$ should be added to the SSF linear predictor to model turning angle with a von Mises distribution with mean 0. In practice, this means that the cosine of turning angle is included as a covariate in the model, and the corresponding selection parameter is equal to the concentration parameter of the distribution.

The above assumed that the selection parameter β_θ associated with $\cos(\theta)$ was strictly positive

(because $\kappa > 0$ and $\beta_\theta = \kappa$). We can relax this assumption by noticing that

$$\kappa \cos(\theta) = -\kappa \cos(\theta - \pi). \tag{8}$$

This implies that, if β_θ is negative, we can interpret $-\beta_\theta$ as the concentration parameter of a von Mises distribution centred on π. A turning angle distribution centred on π corresponds to frequent reversals in direction, which is commonly observed as an artifact of discrete-time observations when animals are inactive.

In summary, including $\cos(\theta)$ as a covariate in the SSF makes it possible to model turning angle with a von Mises distribution, either centred on 0 (if the corresponding selection coefficient β_θ is positive) or centred on π (if β_θ is negative). This covers the vast majority of animal movement scenarios, as other values of the mean turning angle are virtually never used.

B Simulation study

We conducted simulations to verify that our implementation method is able to return the correct parameter values. The basic structure and objective of the simulation study was to: i) simulate a movement track from the HMM-SSF (i.e., with known parameter values), and ii) fit the HMM-SSF with the implementation method described in Section 2.4 and check how well the parameters were estimated.

B.1 Methods

For all simulations, we followed the same algorithm to produce each movement track:

1. Simulate a state sequence $\{S_1, S_2, ..., S_T\}$, with S_1 sample with probabilities given by $\boldsymbol{\delta}$ and $S_2, ..., S_T$ based on $\boldsymbol{\Gamma}$
2. Generate starting location y_1 randomly within the study area Ω
3. To generate each y_t in $\{y_2, y_3, ..., y_T\}$
   (a) Generate many possible endpoints $\{z_1, z_2, ..., z_J\}$ uniformly on a disc with radius r, centred on y_t and evaluate/interpolate relevant covariates.

(b) Select y_{t+1} from the possible endpoints $\{z_1, z_2, ..., z_J\}$ for $j \in 1, 2, ..., J$ with probabilities given by the state-dependent SSF (for the known state $S_t = k$), i.e.,

$$p_j = \frac{\exp\{\boldsymbol{c}(y_t, z_j) \cdot \boldsymbol{\beta}^{(k)}\}}{\sum_{i=1}^{J} \exp\{\boldsymbol{c}(y_t, z_i) \cdot \boldsymbol{\beta}^{(k)}\}} \tag{9}$$

In practice, we generated $J = 10,000$ endpoints within a disc of radius $r = 5$. J was chosen to be very large to reduce the risk of bias arising from the simulation itself. The initial location y_1 was generated from the middle 50% of Ω. Simulations were run for 100 iterations, each for $T = 750$ observations.

We assumed a two-state model meant to represent a generally ideal dataset (i.e., with distinct states and moderate effect sizes), while still being realistic. We assumed that the step lengths followed a gamma distribution (i.e., we included step length and its natural log as covariates) with state-specific means $\mu^{(1)} = 0.5$ and $\mu^{(2)} = 2$ and standard deviations $\sigma^{(1)} = 0.3$ and $\sigma^{(2)} = 1$. We assumed that turning angles followed a von Mises distribution with a mean of 0 (i.e., we included the cosine of the turning angle as a covariate) and state-specific angular concentration parameters $\kappa^{(1)} = 0.25$ and $\kappa^{(2)} = 5$. These parameters can be translated to the corresponding distribution and β parameters following Appendix A. We also considered selection for two additional habitat covariates, which were simulated using a moving average window with spatial autocorrelation parameter ρ (Avgar et al., 2016; Klappstein et al., 2022). Both covariate rasters were simulated with dimensions of 2000×2000 with a resolution of 1. The general raster simulation process was to: i) generate a random value for each raster cell from $[U \sim (0, 1)]$, and ii) determine the covariate value based on a circular moving average window with radius ρ (i.e., a higher ρ indicates more spatial autocorrelation). We defined $\rho_1 = 5$ for covariate 1 and $\rho_2 = 25$ for covariate 2 (Figure 1). In state 1, there was selection for covariate 1 ($\beta = 3$) and selection against covariate 2 ($\beta = -1$). This selection pattern was reversed in state 2, with selection against covariate 1 ($\beta = -1$) and selection for covariate 2 ($\beta = 3$).

We additionally included covariates on the transition probabilities (in addition to the SSF covariates, previously described). We simulated data at a 1-hour time resolution (i.e., 24 steps in a day). Following Towner et al. (2016), we included time of day as a cyclic covariate, such that the

linear predictor in Equation 8 of the main text becomes

$$\eta_{ij}^{(t)} = \begin{cases} \alpha_0^{(ij)} + \alpha_1^{(ij)} \cos\left(\frac{2\pi\tau_t}{24}\right) + \alpha_2^{(ij)} \sin\left(\frac{2\pi\tau_t}{24}\right) & \text{if } i \neq j \\ 0 & \text{otherwise} \end{cases} \tag{10}$$

where τ_t is the time of day at time t. We set our simulation parameters for the two-state model as,

$$\boldsymbol{\alpha} = \begin{pmatrix} \alpha_0^{(12)} & \alpha_0^{(21)} \\ \alpha_1^{(12)} & \alpha_1^{(21)} \\ \alpha_2^{(12)} & \alpha_2^{(21)} \end{pmatrix} = \begin{pmatrix} -2.2 & -2.2 \\ 0.75 & -2.0 \\ 0.1 & 1.0 \end{pmatrix} \tag{11}$$

to represent a higher probability of switching into state 1 during the day and a higher probability of switching into state 2 at night. For all simulations, we fitted the model with $N = 25$ gamma-distributed control steps.

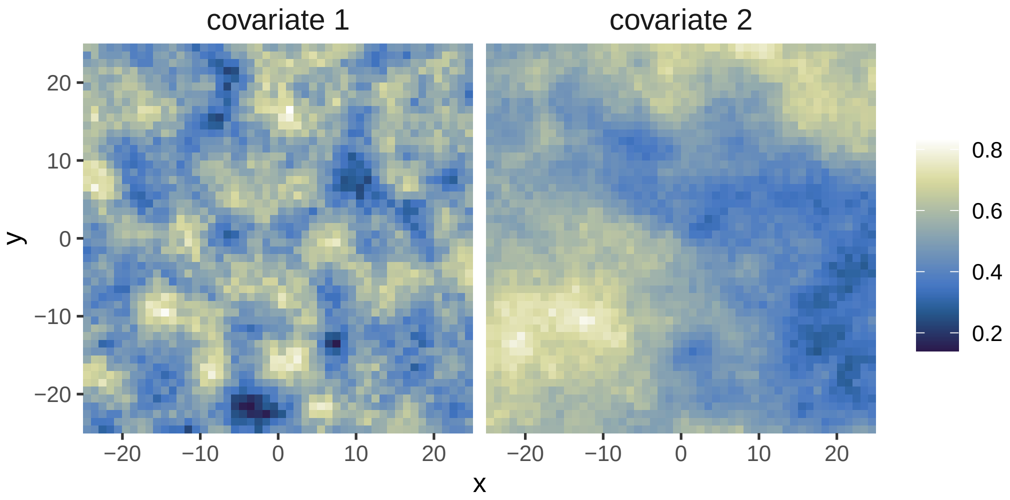

Figure 1: *Example of the simulated habitat covariates, with different spatial autocorrelation parameters ($\rho_1 = 5$ and $\rho_2 = 25$). Shown is the middle cells of the 2000 × 2000 raster, and the color scale indicates the value of the covariate.*

B.2 Results

Generally, movement and habitat selection parameters were estimated well, with the exception of the estimate for covariate 2 in state 1 (Figures 2 and 3). This is most likely because there is high

spatial autocorrelation in covariate 2 and short step lengths in state 1. This seems to cause bias in the direction of the (positive) selection in state 2 because, when the animal switches into state 1, it will tend to already be in an area of high values of covariate 2, and will not be able to move to low values before switching again. The general pattern of transition probabilities in relation to time of day was also captured, but with higher variance on the transition from state 2 to state 1 (Figure 4). States were decoded correctly 98.2% of the time.

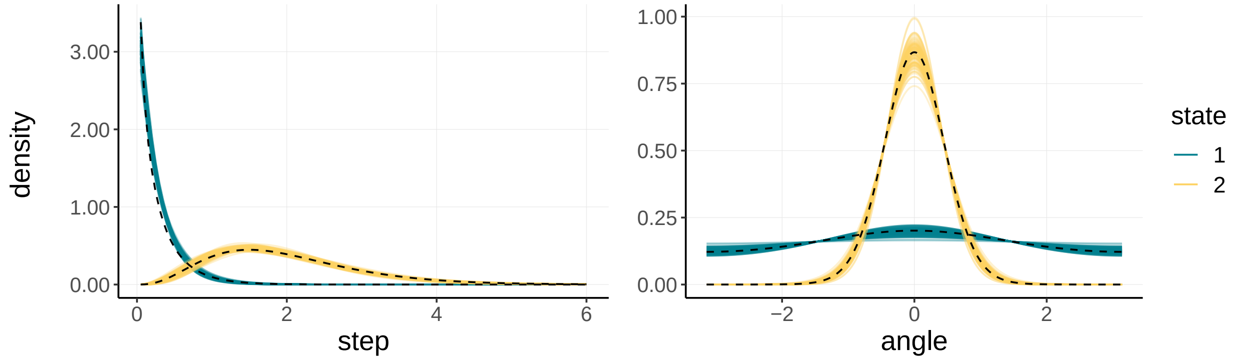

Figure 2: *Estimated step length (top) and turning angle (bottom) distributions for all 100 simulation iterations. True distribution shown as the dashed black line.*

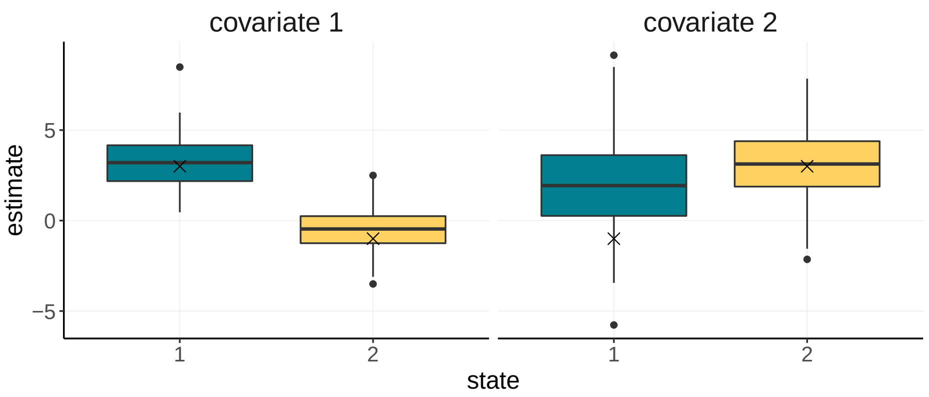

Figure 3: *Boxplot of estimated habitat selection parameters (for covariate 1 and covariate 2) for all 100 simulation iterations. True parameter value shown as the black* $\times$*, and the middle line is the median of the estimates.*

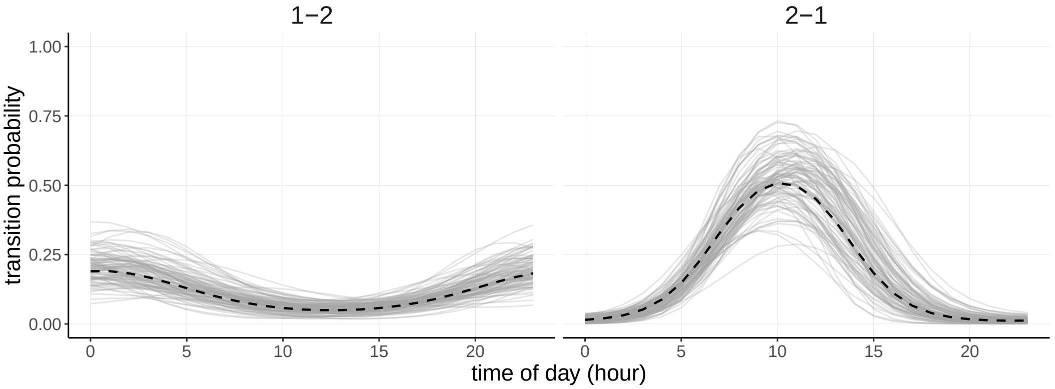

Figure 4: *Estimated transition probabilities as a function of time of day, for transitions from state 1 to 2 (1-2) and from state 2 to 1 (2-1). Each line is one of the 100 simulation iteration estimates and the black dashed line is the true relationship.*

References

Avgar, T., Potts, J. R., Lewis, M. A., and Boyce, M. S. (2016). Integrated step selection analysis: Bridging the gap between resource selection and animal movement. *Methods in Ecology and Evolution*, 7:619–630.

Forester, J., Kyung Im, H., and Rathouz, P. (2009). Accounting for animal movement in estimation of resource selection functions: Sampling and data analysis. *Ecology*, 90(12):3554–3565.

Klappstein, N. J., Potts, J. R., Michelot, T., Börger, L., Pilfold, N. W., Lewis, M. A., and Derocher, A. E. (2022). Energy-based step selection analysis: Modelling the energetic drivers of animal movement and habitat use. *Journal of Animal Ecology*, 91:946–957.

Nicosia, A., Duchesne, T., Rivest, L.-P., and Fortin, D. (2017). A multi-state conditional logistic regression model for the analysis of animal movement. *The Annals of Applied Statistics*, 11(3):1537–1560.

Rhodes, J. R., Mcalpine, C. A., Lunney, D., and Possingham, H. P. (2005). A spatially explicit habitat selection model incorporating home range behavior. *Ecology*, 86(5):1199–1205.

Towner, A. V., Leos-Barajas, V., Langrock, R., Schick, R. S., Smale, M. J., Kaschke, T., Jewell, O. J. D., and Papastamatiou, Y. P. (2016). Sex-specific and individual preferences for hunting strategies in white sharks. *Functional Ecology*, 30:1397–1407.
